# Mechanical Properties and Metallurgical Features of New Green NiTi Reciprocating Instruments

**DOI:** 10.3390/ma13173736

**Published:** 2020-08-24

**Authors:** Luigi Generali, Anastasiya Malovo, Giovanni Bolelli, Alessia Borghi, Giusy Rita Maria La Rosa, Pietro Puddu, Luca Lusvarghi, Alberto Rota, Ugo Consolo, Eugenio Pedullà

**Affiliations:** 1 Department of Surgery, Medicine, Dentistry and Morphological Sciences with Transplant Surgery, Oncology and Regenerative Medicine Relevance (CHIMOMO), University of Modena and Reggio Emilia, 41124 Modena, Italy; malovo2305@gmail.com (A.M.); alessia.borghi@unimore.it (A.B.); ugo.consolo@unimore.it (U.C.); 2 Department of Engineering “Enzo Ferrari” (DIEF), University of Modena and Reggio Emilia, 41125 Modena, Italy; giovanni.bolelli@unimore.it (G.B.); pp.puddu@gmail.com (P.P.); luca.lusvarghi@unimore.it (L.L.); 3 InterMech—MO.RE. Centro Interdipartimentale per la Ricerca Applicata e i Servizi nel Settore della Meccanica Avanzata e della Motoristica, University of Modena and Reggio Emilia, 41125 Modena, Italy; 4 Consorzio Interuniversitario Nazionale per la Scienza e Tecnologia dei Materiali (INSTM), Local Unit: University of Modena and Reggio Emilia, 41125 Modena, Italy; 5 Department of General Surgery and Surgical-Medical Specialties, University of Catania, 95124 Catania, Italy; g_larosa92@live.it (G.R.M.L.R.); eugeniopedulla@gmail.com (E.P.); 6 Department of Physics, Computer Science and Mathematics, University of Modena and Reggio Emilia, 41125 Modena, Italy; alberto.rota@unimore.it

**Keywords:** Auger electron spectroscopy, cyclic fatigue, differential scanning calorimetry, focused ion beam analysis, Procodile

## Abstract

To evaluate the properties of two nickel–titanium (NiTi) reciprocating endodontic instruments (commercially known as Procodile and Reziflow), a total of 40 size 25 and 0.06 taper new Procodile and Reziflow instruments (*n* = 20) were subjected to cyclic fatigue tests (60° angle of curvature, 5-mm radius) at 20 °C and 37 °C and a torsional test based on ISO 3630-1. The fracture surface of each fragment was examined. The morphological, mechanical, chemical, thermal, and phase composition characteristics of the files were investigated by field-emission gun scanning electron microscopy (FEG-SEM) equipped with an energy-dispersive X-ray (EDX) detector, focused ion beam analysis (FIB), micro-Raman spectroscopy, X-ray diffraction (XRD), differential scanning calorimetry (DSC), and Auger electron spectroscopy (AES). Reziflow showed higher cyclic fatigue resistance than Procodile at 37 °C (*p* < 0.05). The maximum torsional strength of Procodile was lower than that of Reziflow (*p* < 0.05). No difference was found between their angular rotations to fracture (*p* > 0.05). SEM, FIB, Micro-Raman, and AES analyses revealed the presence of an Nb/Nb_2_O_5_ coating on the Procodile surface. DSC and XRD analysis confirmed that both files consist of an almost austenitic phase structure at 37 °C. The cyclic fatigue resistance of Procodile and Reziflow significantly decreases upon exposure to body temperature.

## 1. Introduction

Nickel–titanium (NiTi) endodontic instruments, also called files, have numerous advantages, such as greater flexibility and the capacity to follow the root canals without making ledges or perforations [1,2]. Even with these advantages, NiTi instruments can unexpectedly fracture, mainly for flexural or torsional fatigue, and this may negatively influence the treatment prognosis [3,4,5]. Failure may occur under torsional loading, if the file gets locked inside the root canal while the shank still rotates [6], or when the friction between the instrument and canal wall results in a torque above the elastic limit of the alloy. In the latter case, the file shows plastic deformation followed by fracture [4]. Differently, the flexural or cyclic fatigue fracture is determined by continuous cycles of compressive and tensile stresses on a file that is bent while rotating inside a curved canal [7]. Many factors can affect the cyclic fatigue resistance of NiTi rotary files, i.e., the size and taper of the file and the radius and angle of curvature of the root canal [8]. In addition, it has been reported that the cyclic fatigue resistance of NiTi instruments may be negatively influenced by environmental temperature [9,10]. The reciprocating motion consists of an alternating oscillation of the file in both directions, and it may increase the cyclic fatigue resistance of the file [11] compared to the continuously rotated one. This type of motion reduces the risk of fracture, as well as the operating time, even for inexperienced operators [4,12]. Reziflow (Komet, Brasseler GmbH & Co., Lemgo, Germany) are endodontic instruments in traditional NiTi alloy, with an S-cross section and a counterclockwise (CCW) reciprocating motion [13]. Procodile is a new reciprocating NiTi file (Komet, Brasseler GmbH & Co., Lemgo, Germany) with an innovative tapered core that makes it more flexible and efficient due to the increased chip space that guarantees debris evacuation and an optimum preparation, even for curved root canals. Moreover, Procodile has an S-cross section and a distinguishable green coating on its surface. Procodile is designed for work in all reciprocating endo-motors, but it also has a dedicated motor, which has a specific program for this type of instrument [14]. According to the manufacturer’s recommendation, both types of files are conceived for single use [13,14]. To date, no previous studies have investigated the mechanical properties of this new file. However, it could be very useful for clinicians to know more about the mechanical behavior of the new Procodile reciprocating file in order to exploit its possible benefits in clinical practice. Consequently, the aim of this study was to evaluate the surface morphology and phase composition as well as cyclic fatigue resistance and torsional behavior at body and room temperatures of Procodile files and to compare them with Reziflow instruments. The null hypothesis is that there is no difference between the mechanical properties and the metallurgical features of the tested files.

## 2. Materials and Methods 

### 2.1. Sample Size Calculation

Considering a test power of 0.80 (G *Power 3.1.9.2 Software, Heinrich-Heine-Universität Düsseldorf, Düsseldorf, Germany) with α = 0.05 and β = 0.95, 20 instruments per type (*n* = 20) were submitted to a cyclic fatigue test at 20 °C and 37 °C and a torsional resistance test at room temperature. Regarding cross-section area measurement, scanning electron microscopy analysis, electron dispersive X-ray spectroscopy, and focused ion beam analysis, two instruments for each brand were selected. One instrument for each type was selected for micro-Raman spectroscopy and two Procodile files were selected for Auger electron spectroscopy with depth profiling. For X-ray diffraction analysis, three instruments for each brand were included, while multiple (*n* = 2–3) segments of the files between 10 and 15 mg of weight were used for differential scanning calorimetry. 

### 2.2. Cyclic Fatigue Test

Forty new 25-mm long Procodile size 25 and 0.06 taper and Reziflow, with the same taper and tip dimensions, were used. Instruments 25.06 were selected because they are the most common size employed during instrumentation. Stereomicroscopic inspection (SZR-10; Optika, Bergamo, Italy) of each file was preliminarily carried: no sample exhibited defects or deformities. Therefore, all files were included in the study. Cyclic fatigue tests were performed using a customized device, which has previously been described in [5,15]. Briefly, artificial canals were machined in a stainless steel block, which was mounted onto a mobile platform performing a linear reciprocating motion. The file was operated by an electric handpiece, which was fixed into a support unit. This unit was designed to guarantee repeatability in the positioning of the handpiece and the inclination of the file with respect to the artificial canal. For this study, instruments were inserted in the standard position (0°) into a 16-mm long canal with a 60° angle and 5 mm radius of curvature. The center of the curvature was placed 5 mm from the tip of the tested file. The canal had the same taper and macroscopic geometry of the tested files, but its diameter was 150 µm larger. A thermostatic resistance was associated to the customized device in order to allow temperature adjustment inside the artificial canal. The temperature was kept constant during the test using a thermocouple applied to the artificial canal, which activates or deactivates the thermostatic resistance when the temperature decreases or reaches the preset one, respectively. In particular, the instruments were tested at room (20 ± 1 °C) and body temperature (37 ± 1 °C). The tested files were activated by a 6:1 reduction handpiece (Sirona Dental Systems GmbH, Bensheim, Germany) powered by an endodontic motor (Silver Reciproc, VDW) using the preset programs “Reciproc ALL” to activate both files. According to the manufacturer, the Reciproc mode has a 300 rpm (rotations per minute) speed [16]. The canal was lubricated with synthetic oil (Super Oil; Singer Co Ltd., Elizabethport, NJ, USA) to reduce the friction of the file against the canal walls. All instruments were rotated until fracture. For each file, the time to fracture (TtF), from the start of the test until the separation was discerned visually and/or audibly, was recorded using a digital chronometer with an accuracy of ± 0.1 s. The tests were also video-recorded to control the time of files separation, trying to avoid human errors. A digital microcalliper (Mitutoyo Italiana srl, Lainate, Italy) was used for measuring the length of the separated file tip.

### 2.3. Torsional Fatigue Test

In the torsional tests, a custom-made device (ISO 3630-1) was used for evaluating the mean ultimate torsional strength and angle of rotation of the tested instruments. The device has been described previously [17,18]. The file was clamped at 3 mm from the tip and connected to a torque sensor, whereas at the other end, the shaft of the file was connected to a stepper motor and rotated counterclockwise at 2 rpm until fracture. The peak torque (N∙cm) and corresponding angular rotation at failure (°) were measured using a torsiometer (Sabri Dental Enterprises, Downers Grove, IL, USA). Tests were carried out at room temperature (20 ± 1 °C).

Fracture surfaces were inspected by field-emission scanning electron microscopy (Nova NanoSEM 450; FEI, Eindhoven, NL, USA) at 500× and 6000× magnifications to reveal failure mechanisms.

### 2.4. Cross-Section Area Measurement

Two new Procodile and two new ReziFlow were embedded in epoxy resin in vertical position and cut using a saw microtome (Leica SP1600; Leica Microsystem Nussloch GmbH, Nussloch, Germany) under continuous water spraying at 3, 4, 5, 6, and 8 mm from the tip. Each cross-section was observed under a field-emission gun scanning electron microscopy (FEG-SEM) microscope (Nova NanoSEM 450; FEI, Eindhoven, NL, USA) at 378× magnification. The areas of the file sections were calculated in µm^2^ using Fiji software (National Institutes of Health, Bethesda, MD, USA). 

### 2.5. Scanning Electron Microscopy Equipped with Electron-Dispersive X-ray Spectroscopy and Focused Ion Beam Analysis

Two new files for each instrument type were observed by scanning electron microscopy (SEM: Nova NanoSEM 450 equipped with a field emission gun—FEG—source: FEI—ThermoFisher Scientific, Hillsboro, OR, USA). Samples were mounted on aluminum specimen stubs using a graphite-based conductive tape, with no further preparation, in order to observe their pristine surface state. Qualitative chemical analyses were performed using an energy-dispersive X-ray (EDX) detector (Quantax-200 system with XFlash 6/10 Si-drift detector: Bruker Corp., Billerica, MA, USA).

Two new Procodile files were sectioned in-situ using a focused ion beam (FIB) device for investigating the possible existence and nature of any surface layer without introducing artificial damage during a conventional metallographic (mechanical cutting/polishing) procedure. Specifically, a FIB+SEM apparatus (Strata™ DB235: FEI) was employed to perform ion milling and imaging in a single procedure, which has been described in [19]. Sections were made on a relatively flat region along the working part of the files. A protective platinum layer was first deposited on the area of interest; then, a 5-µm deep ditch was milled with high ion-beam current (7 nA), and its side was smoothed with a lower beam current (300 pA). The cross-section was finally observed using the SEM column with a tilt angle of 52°.

### 2.6. Micro-Raman Spectroscopy

Micro-Raman spectra were acquired on the surface of one new Procodile and one new Reziflow instrument using a LabRAM (Horiba—Jobin Yvon, Kyoto, Japan) device. The excitation source was a solid-state laser (wavelength: 532 nm). This focused onto relatively flat portions of the working part using 50× and 100× objectives. Spectra were employed in order to appraise the nature of any non-metallic surface layer on the samples.

### 2.7. X-ray Diffraction Analysis

The working parts of 3 new Procodile and Reziflow files were mechanically removed from the shaft, embedded in epoxy resin, hardened at room temperature, and ground with SiC-based abrasive papers of increasingly fine abrasive size (from P400 to P2500) in order to expose their section. Resin-mounted, ground sections were eventually polished using a polycrystalline diamond (3 µm average particle size) slurry and a ≈60 nm colloidal silica suspension (Oxide Polishing Suspension—OPS) in order to remove any mechanically altered surface layer. X-ray diffraction (XRD) analysis was performed at room temperature (≈25 °C) on the polished sections, using Ni-filtered Cu–Kα radiation. The X-ray source was operated at 40 mA filament current and 40 kV acceleration voltage (X’Pert PRO diffractometer, PANAlytical, Almelo, NL, USA). Patterns were acquired over a 30° < 2θ < 100° range with a step size of 0.017° and a counting time of 1100 s/step. Qualitative phase identification was carried out by comparison to the ICDD JCPDF-2 database using the X’Pert Highscore Plus software (PANAlytical) [19,20].

### 2.8. Differential Scanning Calorimetry

The same procedure already reported in the literature [19] was employed for assessing the transformation behavior of Procodile and Reziflow instruments during heating and cooling through differential scanning calorimetry (DSC—Q2000, TA Instrument, New Castle, DE, USA). The working part of new instruments was mechanically split into smaller portions and loaded into an Al crucible up to a total sample mass comprised between 10 and 15 mg. Such samples were subjected to 2 successive heating/cooling thermal cycles at a rate of 5 °C min^−1^ in flowing N_2_ atmosphere, within a temperature range between −40 °C and +110 °C [19,20], starting from room temperature (20 °C). Plots were analyzed using Universal Analysis 2000 (TA Instrument) software to obtain onset, peak, and end temperatures of phase transformation and the associated enthalpy changes (ΔH). Specifically, the intersection between the extrapolation of the baseline and the maximum gradient line of the lambda-type DSC curve gave the transformation temperatures [21].

### 2.9. Auger Electron Spectroscopy with Depth Profiling

Auger electron spectroscopy (AES) with depth profiling was conducted on two Procodile instruments for determining the chemical composition across the surface layers, up to a depth of a few micrometers. A portion of the working area of the Procodile instrument was mechanically cut; then, it was mounted onto an aluminum specimen stub using a graphite-based conductive tape for SEM observations.

A coaxial CMA PHI 15-110a Auger electron spectrometer equipped with a SPECS PU IQE 12/38 Ar^+^ ion gun was employed. Spectra were acquired using a primary electron beam energy of 3 keV, with a beam current of approximately 500 nA on a sample area of approximately 50 × 50 µm^2^. The surface of the sample was stepwise milled through the ion gun, which was operated at an ion beam energy of 3 keV and a beam current density of 200 µA/cm^2^ over an area of 0.5 × 0.5 mm^2^. Through stepwise removal of a controlled thickness, Auger spectra reflect a through-thickness compositional profile across the near-surface region of the Procodile sample, in order to determine the composition of its distinctive green coating.

### 2.10. Statistical Analysis 

As regards cyclic and torsional fatigue tests, data were subjected to the Shapiro–Wilk test to characterize their normality, and because they showed a normal distribution, they were statistically analyzed using two-way analysis of variance (ANOVA) and the Tukey multiple comparison post hoc test (Prism 7.0; GraphPad Software, Inc, La Jolla, CA, USA) with the significance level established at 5% (*p* < 0.05). 

## 3. Results

### 3.1. Cyclic Fatigue Test

**Figure 1:**
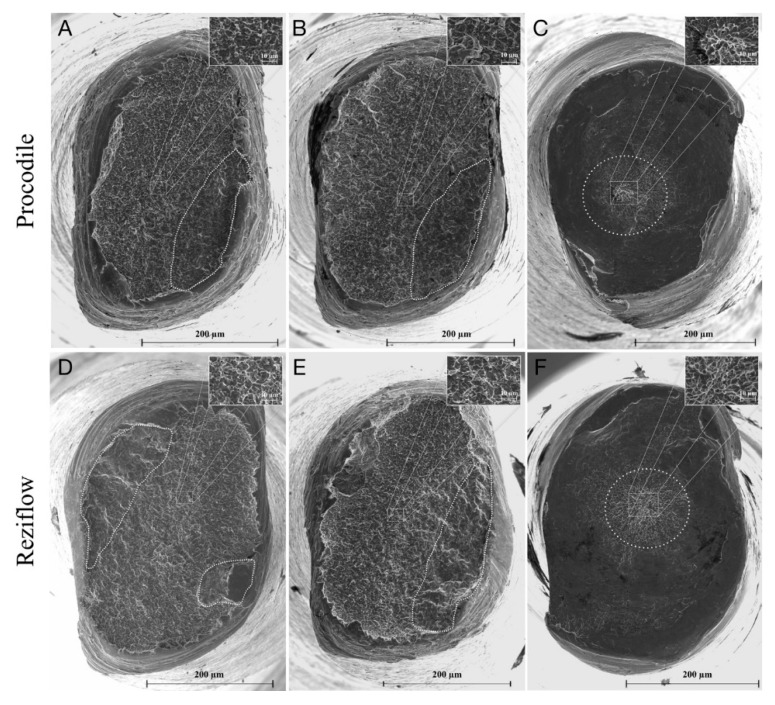
Field-emission gun scanning electron microscopy (FEG-SEM) micrographs of fracture surface (axial view) of Procodile (**A**–**C**) and Reziflow (**D**–**F**) after the cyclic fatigue test at 20° C (**A**,**D**) and at 37 °C (**B**,**E**) and after torsional test (**C**,**F**). Dotted lines (**A**,**B**,**D**,**E**) highlight the crack propagation area, and insets show magnified details of dimples in the overload fracture area. The dotted circles (**C**,**F**) indicate concentric abrasion marks, and insets show magnified details of skewed dimples near the center of the section.

Cyclic fatigue testing of all instruments at 37 °C significantly reduced their TtF compared with the ones fractured at 20 °C (*p* < 0.05), Table 1. Comparing the two instruments, there was no significant difference between Procodile and Reziflow at 20 °C (*p* > 0.05), while Reziflow exhibited a higher TtF than Procodile at 37 °C (*p* < 0.05). The mean length of the fractured fragment (5.02 mm) was not significantly different for all of the instruments tested (*p* > 0.05). Figure 1 shows the fracture morphologies, where crack initiation/propagation areas and final fracture zones can be identified.

### 3.2. Torsional Fatigue Test

The maximum torsional strength of Procodile was lower than that of Reziflow (*p* < 0.05), as shown in Table 1, whereas their angular rotation to fracture did not differ significantly (*p* > 0.05). Fracture surface morphologies are analogous for both file types. They are characterized by concentric abrasion marks and fibrous dimple marks at the center of rotation, which is typical of torsional failure (Figure 1).

### 3.3. Cross-Section Area Measurement

Procodile and Reziflow have the same S cross-section, the same taper, and the same tip diameter, but they have different core areas. The Procodile instruments have a variable core area from the tip to the shaft with the manufacturer aim to reduce the thickness of the core for the optimum preparation of the curved root canals and a better dentin removal due to an enlarged chip space. The areas of the cross-sections at 3, 4, 5, 6, and 8 mm of the Procodile and Reziflow files are summarized in Table 2.

### 3.4. FEG-SEM/EDS and FIB Analyses 

**Figure 2:**
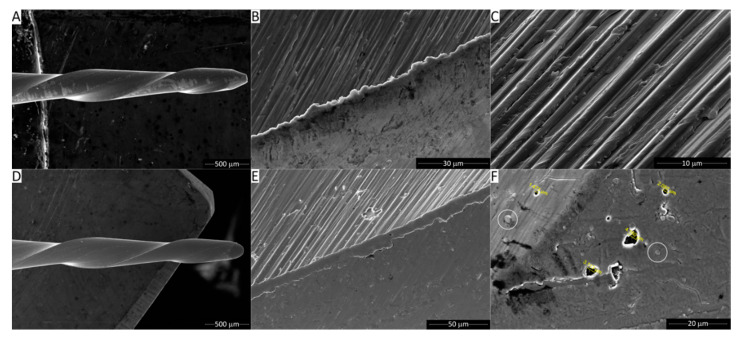
Secondary electron SEM micrographs showing overviews (**A**,**D**) and details (**B**,**C**,**E**,**F**) of the working area of the Reziflow (**A**–**C**) and Procodile (**D**–**F**) files. The circles in panel F indicate clusters.

**Figure 3:**
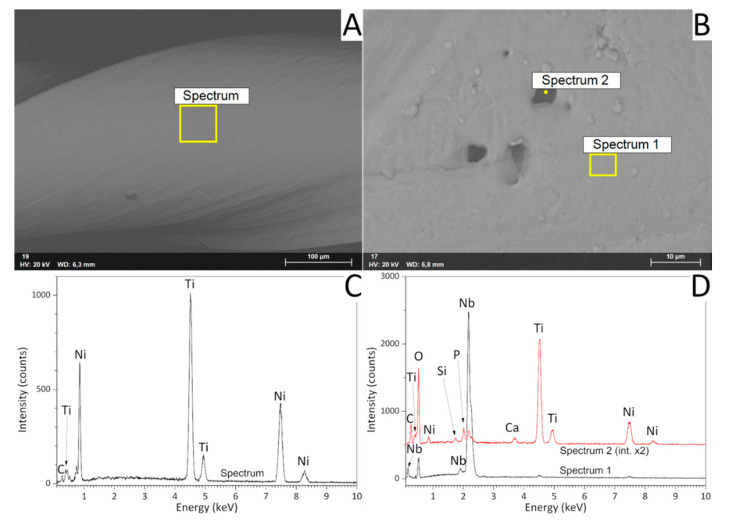
Backscattered electron SEM micrographs (**A**,**B**) and corresponding energy-dispersive X-ray (EDX) spectra (**C**,**D**) acquired on the working area of the Reziflow (**A**,**C**) and Procodile (**B**,**D**) instruments.

The outer surface of the Reziflow instrument (Figure 2A–C) revealed the characteristic grooves resulting from the micro-milling process. EDX spectra only revealed the characteristic elements of the NiTi alloy (Figure 3A,C). The Procodile instrument looked macroscopically similar (Figure 2D). However, in detail, it appeared as a thin surface film covered the machining grooves (Figure 2E). The surface film contained some micrometric pores and defects (the sizes of some of which are shown in Figure 2F); moreover, it also displayed equally small clusters (some of which are encircled in Figure 2F). This morphology is typical of micrometer-thin coatings deposited by physical vapor deposition (PVD) processes, especially those deposited using sputtering sources [22]. EDX spectra clarified that the surface film was based on Nb and contained some oxygen (Figure 3B,D—Spectrum 1). The weak EDX signal from Ni and Ti testified to the rather low film thickness, since the X-ray generation region could likely extend to the underlying NiTi alloy. Spectra acquired at the bottom of the pores revealed additional peaks belonging to Si, Ca, and P. Whilst the presence of Si and Ca might be ascribed to foreign contaminants having entered the defects, this might not be the case for P. Therefore, it is possible that the surface film actually consists of at least two layers, the bottom one containing P.

**Figure 4:**
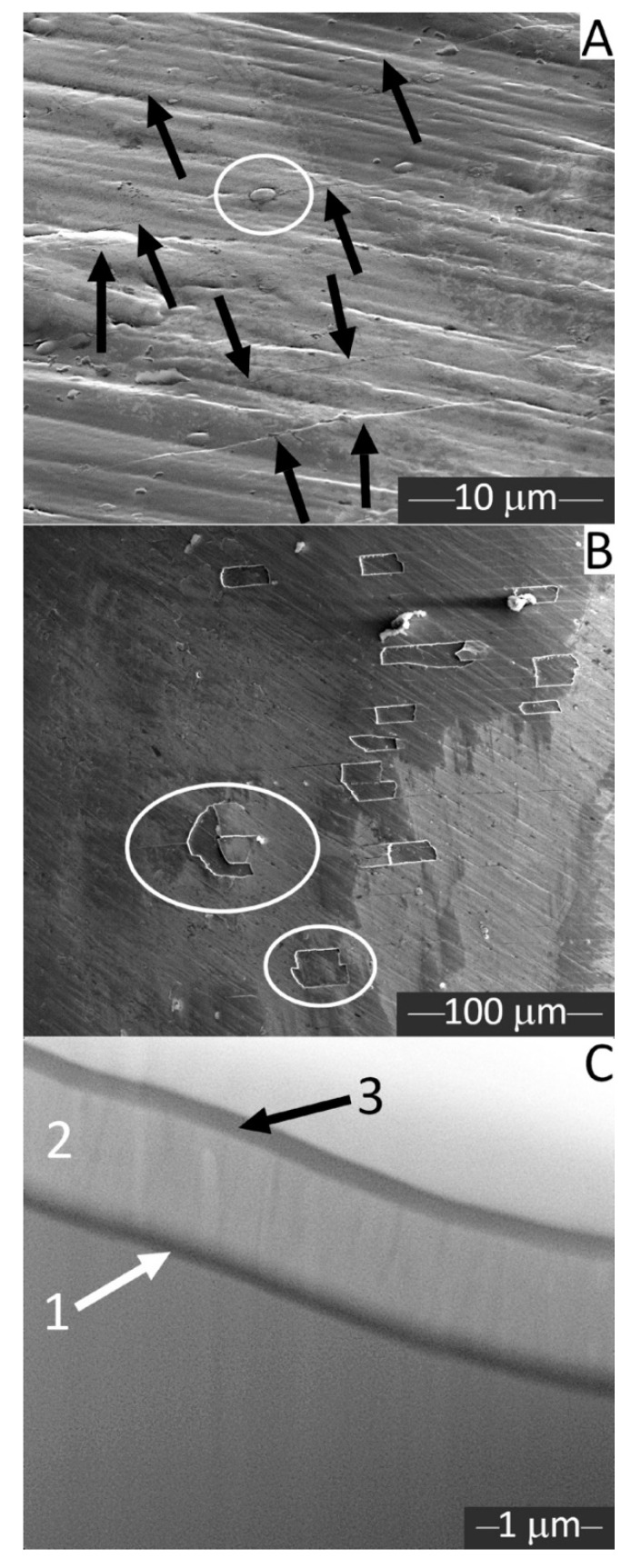
Secondary electron SEM micrographs: detail (**A**) and overview (**B**) of the surface of a Procodile file, imaged under a tilt angle of 52° (arrows: microcracks, circle in panel A = cluster; circles in panel B = delaminations), and cross-section of the Procodile file (**C**) obtained by in situ FIB machining (numbers indicate the distinct layers composing the surface coating of the Procodile file).

A highly detailed SEM micrograph acquired on a Procodile sample tilted at 52° inside the dual-beam FIB+SEM equipment (Figure 4A), besides confirming the presence of tiny clusters (see circle), also revealed numerous cracks across the film (see arrows). The cracks were all oriented perpendicular to the direction of the instrument axis. In some areas of the working region of the file (e.g., see Figure 4B), the cracks evolved into larger delaminations of the film.

Cross-sections obtained in situ by FIB milling/polishing (Figure 4C) confirmed that the film was quite thin. Its thickness, calculated by image analysis from SEM micrographs, was 2.2 ± 0.1 µm. They also confirmed that the film consisted of a three-layer system, with a thicker and brighter intermediate layer (label 2) and thinner, darker layers at the bottom (label 1) and on top (label 3). The bottom layer was likely the one containing P, based on the EDX spectra described previously.

### 3.5. Micro-Raman Spectroscopy

**Figure 5:**
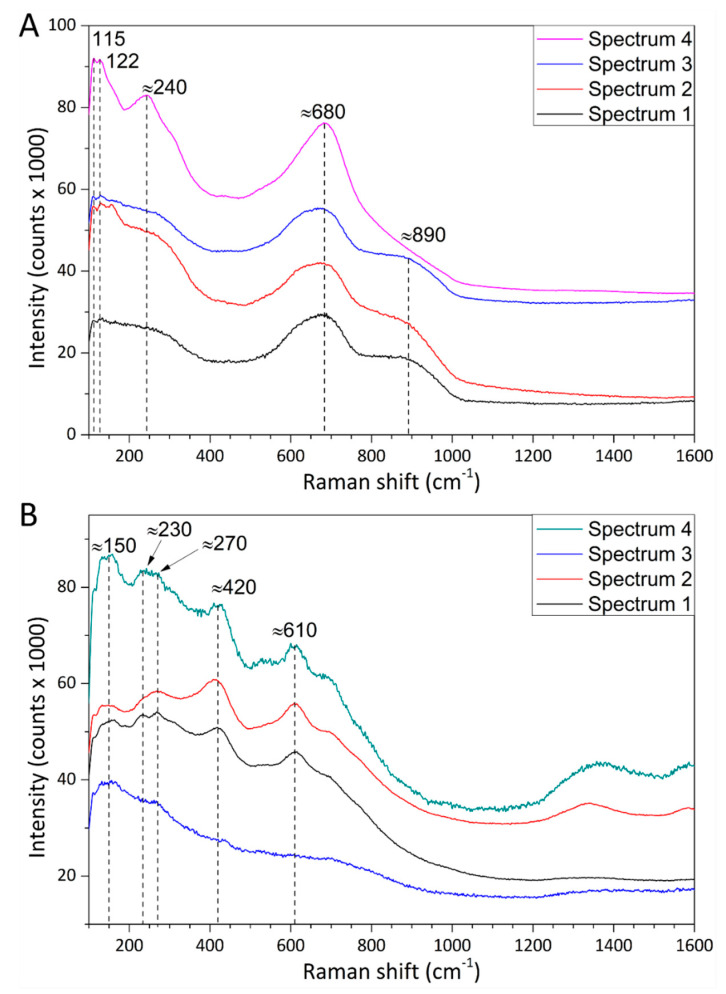
Micro-Raman spectra acquired on the surface of the working parts of the Procodile (**A**) and Reziflow (**B**) files. Spectra 1–4 were acquired at distinct, randomly chosen positions.

The Procodile sample exhibited broad Raman peaks at approximately 120 cm^−1^ and 250 cm^−1^, and a broad band at 660–690 cm^−1^ with a shoulder around 870 cm^−1^ (Figure 5A). This spectrum matches with those listed in the literature for amorphous and/or orthorhombic Nb_2_O_5_ [23,24]. Coupling this information with the cross-sectional morphology of the surface film seen in Figure 4C and its EDX spectrum (Figure 3D—Spectrum 1), it is inferred that the thin, darker top layer of the Nb-based film consisted of Nb_2_O_5_. Instead, the underlying, thicker, and brighter layer probably consisted of pure Nb, whilst no further information could be gained on the bottom layer. The oxidized surface layer was probably responsible for the peculiar green color of the Procodile instruments. This might be due to interferential coloring effects, such as the interference colors reported for anodized niobium, since the thickness of the oxide layer (new hundreds of nanometers, Figure 4) is comparable to the wavelength of visible light [25,26,27]. Alternatively, the color might also be affected by small amounts of doping elements, which are below the detection limit of the EDX technique.

On the other hand, the Reziflow sample exhibited weak Raman peaks mainly located around 120 cm^−1^, 230 cm^−1^, 420 cm^−1^, and 610 cm^−1^ (Figure 5B), which could be assigned to the rutile polymorph of TiO_2_ [28]. Therefore, it was inferred that the uncoated NiTi surface of the Reziflow file was slightly oxidized. Oxidation might have occurred due to local overheating during turning or during a subsequent heat treatment. As it was observed in a previous study on other kinds of NiTi instruments [19], indeed, the affinity of Ti toward oxygen is so high that even an extremely low oxygen partial pressure during a heat treatment carried out in vacuum or controlled atmosphere is enough to cause oxide formation.

### 3.6. Auger Electron Spectroscopy (AES)

**Figure 6:**
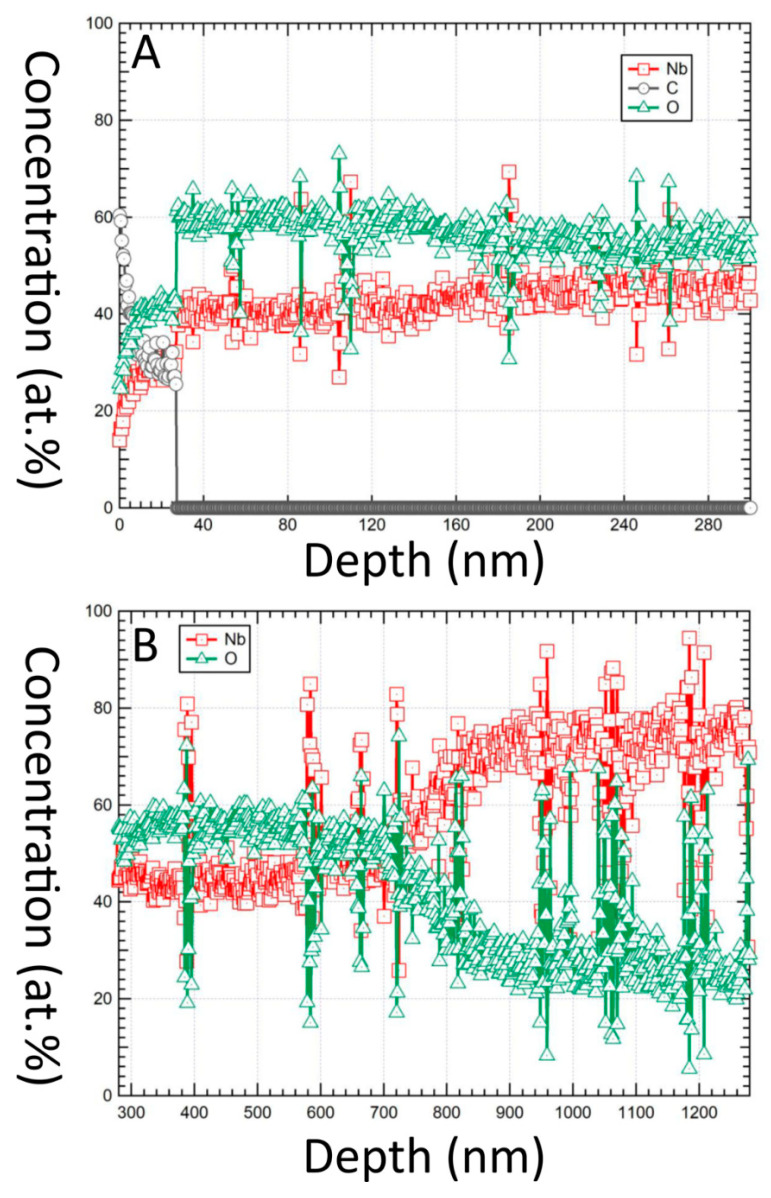
Compositional depth profiles of the Procodile sample, obtained by Auger electron spectroscopy (AES) coupled to Ar^+^ ion bombardment. Profile in the near-surface region (**A**) and at depth ≥300 nm (**B**).

The through-thickness compositional profile of a Procodile file, investigated by AES with Ar^+^ ion bombardment, revealed that below a ≈30-nm thick C-rich layer due to unavoidable surface contamination of a sample exposed to the atmosphere (Figure 6A), Nb and O were the only constituents in detectable amount up to a depth of ≈450–500 nm (Figure 6B). This was consistent with FIB/SEM and micro-Raman analyses, which showed that the coating onto the Procodile file has an Nb_2_O_5_ top layer, which is some hundreds of nanometers thick. At larger depths, the signal of oxygen decreased and that of Nb increased (Figure 6B). Therefore, it was inferred that the underlying coating layer consisted of metallic Nb, which is consistent with its brighter contrast in SEM micrographs. The oxygen signal never dropped to zero due to an ion mixing effect; hence, the detection of residual oxygen at depths greater than approximately 500 nm should be regarded as a measurement artefact.

### 3.7. X-ray Diffraction Analysis

**Figure 7:**
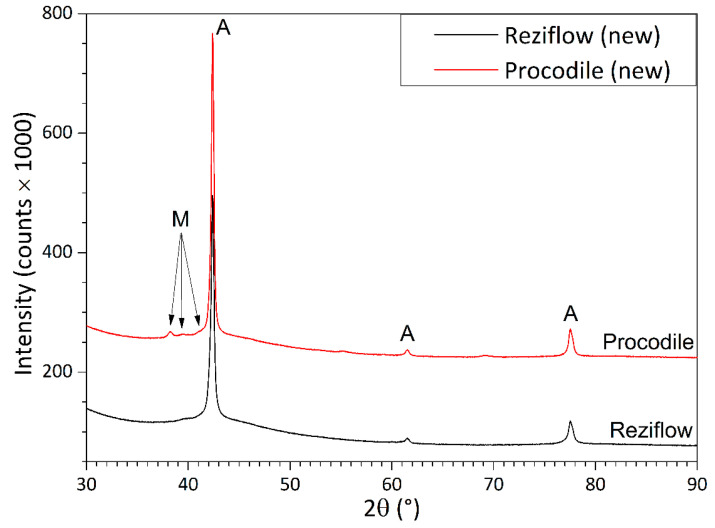
X-ray diffraction (XRD) patterns acquired on the polished longitudinal sections of the Procodile and Reziflow files. Legend: A = austenite (JCPDF 18-899), M = martensite (JCPDF 35-1281).

XRD patterns (Figure 7) revealed that both Procodile and Reziflow files consisted almost exclusively of single-phase austenite at room temperature. Very weak peaks of martensite appear in the pattern of Procodile; however, based on their intensity, the amount of martensite in this sample is minimal.

### 3.8. Differential Scanning Calorimetry

**Figure 8:**
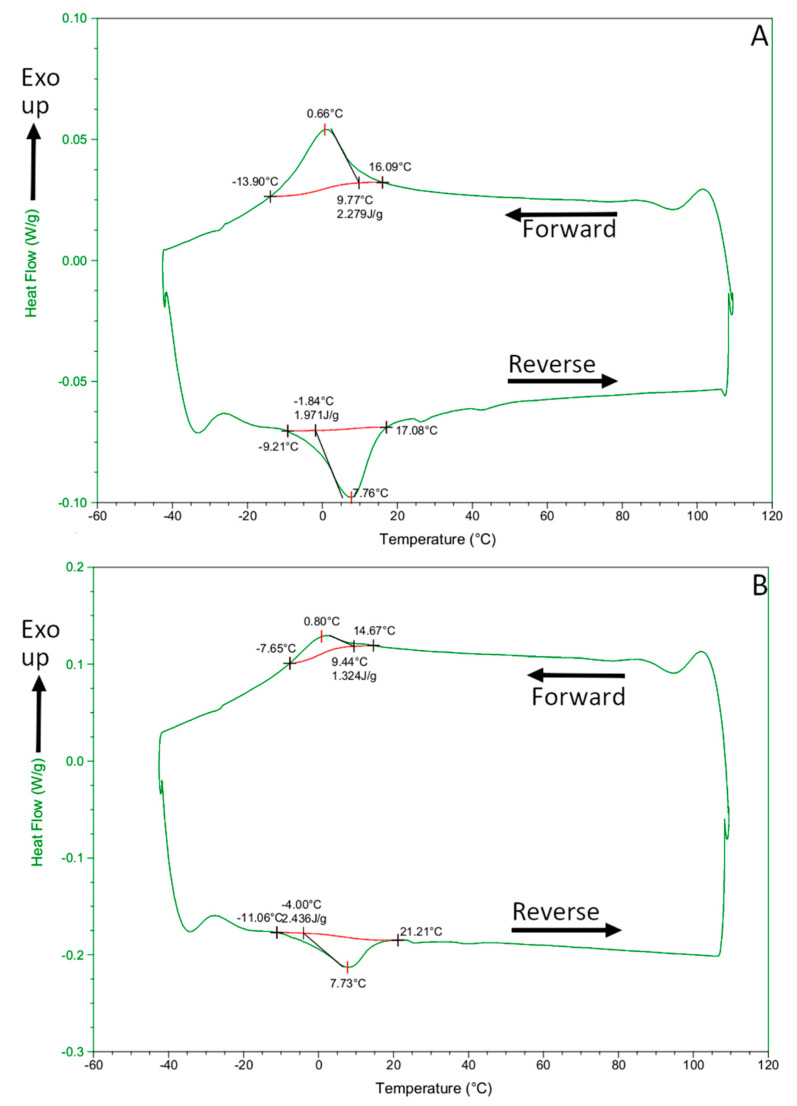
Differential scanning calorimetry (DSC) curves during forward and reverse thermal cycles on Reziflow (**A**) and Procodile (**B**) samples.

DSC curves showed that the forward (cooling) and reverse (heating) transition ranges of both Reziflow (Figure 8A) and Procodile (Figure 8B) instruments were very similar. All transition temperatures are summarized in Table 3: following established conventions in the literature, “Mf” and “Ms” stand for the beginning and end of the martensitic transformation during the forward transition; “As” and “Af” stand for the beginning and end of the austenitic transformation during the reverse transition. All transition ranges occurred below room temperature, which is consistent with XRD patterns showing fully or almost fully austenitic structures at room temperature. Specifically, both the Mf/Ms range during the forward (austenite → martensite) transition and the As/Af range during the reverse (martensite → austenite) transition occurred approximately around −10 °C/+15 °C.

## 4. Discussion

In this study, the mechanical properties of Procodile and Reziflow NiTi instruments at room (20 ± 1 °C) and body (37 ± 1 °C) temperatures were investigated. A major limit of many in vitro tests that examine the mechanical properties of NiTi rotary instruments is the presence of confounding factors such as material properties or geometrical features. Moreover, these variables are also brand-specific, making it hard to evaluate the impact that a single factor has on the mechanical properties of the instrument. However, Procodile and Reziflow files are similar in design (S-cross section) and are both used in reciprocating motion. They differ only for the variably tapered core and the external green coating of Procodile files. 

Cyclic fatigue results were recorded in TtF because time is much easier, reliable, and clinically understandable for the operator than the number of cycles to fracture, especially for the reciprocating instruments, considering that there is a difference between actual kinematics values and manufacturers’ declared values for Reciproc modes, as previously reported [29].

According to our results, both instruments exhibited significantly lower TtF at 37 °C. These outcomes agree with previous studies that reported how high temperatures influence the cyclic fatigue of NiTi files negatively [30,31]. 

At room temperature, an equiatomic NiTi alloy is characterized by an austenite phase unless it is subjected to suitable thermal or thermo-mechanical treatments [32]. Austenitic NiTi files can exhibit superelasticity. Stress-induced martensite formation allows the material to accommodate large strains without permanent plastic deformation (superelasticity), because all the characteristic temperatures (M_s_, M_f_, A_s_, A_f_) increase with stress, until Ms and also Mf become greater than the operating temperature. The effect is fully reversible: when stress is removed, the transformation temperatures decrease again, and the austenite phase is recovered, restoring the original shape [33]. Superelastic instruments can be flexible enough to adapt to the complex shape of root canals. However, proprietary heat treatment procedures that turn the NiTi alloy into multi-phase mixtures of austenite, martensite, and/or rhombohedral R-phase at body temperature [20] reportedly produce files with even better flexibility [32]. Austenitic files are comparatively stiffer and more brittle [32]. In the present study, DSC results showed that the Af temperature of Procodile was 21.21 °C and the Af temperature of Reziflow was 17.08 °C. XRD patterns confirmed that both files consist almost exclusively of austenite, even at room temperature. These results indicated that both instruments are almost completely austenitic even at the intracanal temperature [34,35]. In addition, no difference was observed between the fatigue resistance of Procodile and Reziflow at 20 °C, while at 37 °C, Reziflow exhibited higher TtF. It is difficult to explain why the cyclic fatigue resistance of Procodile was affected more by higher temperature. One factor could be its reduced cross-sectional area compared to Reziflow at 5 mm from the tip corresponding to the point of cyclic fatigue fracture (Procodile = 156,393 µm^2^ and Reziflow = 189,811 µm^2^). Indeed, crack propagation from the origin to the final overload zone of fracture could be more rapid because of the lower cross-sectional area [36]. Another possible factor might be the slightly higher M_s_ temperature of Reziflow (Table 3): this could imply that superelastic transition to martensite begins at lower strain levels, i.e., higher amounts of martensite are produced at the same applied strain. Since martensite is reportedly more flexible than austenite and offers improved fatigue resistance [33,37], a greater amount of martensite formed by superelastic deformation might be beneficial to enhance the TtF of Reziflow. Finally, Procodile files have a coating which, as shown in Figure 4, tends to crack and chip when deformed (this is discussed in more detail hereafter). Such defects can facilitate the nucleation of fatigue failure.

The simulated canals were geometrically similar to the tested files to eliminate the slight deviations in file positioning, which can influence the cyclic fatigue behavior. According to previous studies, fracture occurred close to the maximum curvature, in the range of 4.52–4.99 mm from the file tip, showing that maximum bending stress occurred at similar locations in each condition. This confirmed the precise positioning of the file [38,39]. 

The manufacturer has developed a special movement pattern (ReFlex smart, ReFlex dynamic) for the use of the instruments, which is adapted to the Procodile files, with a corresponding motor (Endopilot, Schlumbohm, Brockstedt, Germany). In this study, the Endopilot motor was not used, as the manufacturer declares that the Procodile files can be used on all the most common motors that develop a reciprocating movement, and this has allowed comparing the cyclic fatigue of the two instruments by eliminating the variable of two different movements. In a future study, it would be interesting to evaluate the cyclical fatigue of Procodile with the Reflex movement generated by the dedicated motor, as the results would probably be even better.

For reciprocating instruments, there is no agreement on the number of cycles to failure (NCF), because there are different reciprocating angles from one file to the other [40], and the cyclic fatigue was expressed as time to fracture (TtF) [19]. Torsional tests in this study showed no differences in the angular rotation between Reziflow and Procodile, while the failure torque of Reziflow was significantly higher. These findings are reasonable, because these instruments are made by the same alloy, but Reziflow at 3 mm from the tip presented a higher cross-sectional area than Procodile, explaining the higher torsional resistance observed for Reziflow [17]. SEM analysis showed typical fractographic features: striations in the crack propagation area and dimples in the overload fracture are in case of cyclic fatigue failure, while there are fibrous dimple marks in the center of the section for torsional failure, without relevant differences between the two tested files [17].

An interesting note concerns the characteristic “green” coating on the Procodile instruments. SEM, FIB, Micro-Raman, and AES analyses revealed a complex, three-layer system, the main constituent of which was a metallic Nb layer with a thin Nb_2_O_5_ outer layer. Based on its peculiar surface morphology, this coating was probably obtained by a physical vacuum deposition process. Nb and Nb_2_O_5_ are known as biocompatible materials, due to their inertness [41]. Specifically, Nb_2_O_5_ has high chemical stability; therefore, metallic Nb is also inert in the body environment, as its surface can be passivated by a thin, dense Nb_2_O_5_ film. The latter can either be formed by spontaneous exposure to the environment, or its formation can be enhanced by some dedicated oxidation treatment. Nb is mostly used as a coating onto other metallic surfaces, in order to take advantage of its corrosion resistance and chemical inertness whilst avoiding the drawbacks due to its poor mechanical properties in bulk form [41]. Based on these considerations, an Nb/Nb_2_O_5_ coating onto the NiTi files would appear to be a reasonable choice. However, neither Nb nor Nb_2_O_5_ are superelastic; hence, they do not possess the same flexibility of the NiTi instrument. As a result, when a flexible NiTi instrument is deformed in clinical use or even through casual handling, the film tends to crack and spall off, as revealed by SEM inspection. Delamination and crack formation defeat the purpose of a protective Nb/Nb_2_O_5_ coating. Moreover, they engender the risk of releasing debris (albeit very fine) inside the root canal during clinical use. It is ultimately concluded that the deposition of an Nb-based surface coating on the NiTi instruments is probably not warranted and should preferably be avoided. Indeed, NiTi itself has very good corrosion resistance and needs not be protected further for endodontic applications. It passivates through the formation of a TiO_2_-based surface layer, as revealed by the micro-Raman spectra of the Reziflow instrument.

Limited to the experimental conditions, the outcomes of the present investigation could be relevant because they provide clinicians with an indication of the possible clinical applications and limits of these files. In particular, the clinical implication of higher cyclic fatigue resistance at body temperature of Reziflow files compared to Procodile files could be the suggestion to use Reziflow files in curved canals in which the flexural stress is high. Moreover, Reziflow files could be also used in constricted canals that might induce higher torsional load stresses. On the other hand, the two files exhibited similar values in terms of angle of rotation and consequently presented similar times before torsional fracture. Interestingly, the green coating of Procodile does not seem to give advantages in terms of corrosion resistance, while it could be disadvantageous, causing crack formation and releasing debris during clinical use.

## 5. Conclusions

On the basis of the results obtained, the null hypothesis was rejected. Procodile and Reziflow files revealed an almost austenitic phase structure at room and body temperature as confirmed by DSC and XRD analyses. Procodile and Reziflow have the same S-cross section but different area; in fact, Procodile revealed a variable core area, and this might influence the torsional resistance of the files. The instruments exhibited also similar values of cyclic fatigue resistance at 20°, but at 37°, the Procodile resistance was lower than the Reziflow one. The main difference, besides the cross-sectional area, is the color of the two files. The green color of Procodile was due to a complex Nb/Nb_2_O_5_ coating that was not present on Reziflow. The Nb/Nb_2_O_5_ coating does not seem to give advantages to the files, because NiTi has already a good corrosion resistance, so it does not need to be protected, and the coating is not as superelastic as NiTi alloy, so it tends to crack.

## Figures and Tables

**Table 1:** Mean ± standard deviation of the time to fracture (TtF), torque (N∙cm), and angle of rotation (°) of the tested instruments.

Instrument	TtF (s) 20 °C	TtF (s) 37 °C	Torque (N∙cm)	Angle of Rotation (°)
Procodile	186 ^a1^ ± 62	126 ^b1^ ± 33	0.70 ^1^ ± 0.08	323.5 ^1^ ± 30.4
Reziflow	222 ^a1^ ± 32	174 ^b2^ ± 37	1.12 ^2^ ± 0.24	302.9 ^1^ ± 53.9

Different superscript letters in the same row indicate statistically significant differences in TtF between the same instruments at 20 °C and 37 °C (*p* < 0.05). Different superscript numbers in the same column indicate statistically significant differences in TtF, torque, and angle of rotation between the different brands of files (*p* < 0.05).

**Table 2:** Cross-section area (µm^2^) of Procodile and Reziflow at different levels from the tip to the shaft.

Instrument	3 mm	4 mm	5 mm	6 mm	8 mm
Procodile	101,346	124,296	156,393	189,693	243,813
Reziflow	123,393	149,159	189,811	231,245	308,145

**Table 3:** Average phase transformation temperature and enthalpy changes (∆H) of the tested instruments.

	Cooling			Heating		
	M_s_ (°C)	M_f_ (°C)	∆H (J/g)	A_s_ (°C)	A_f_ (°C)	∆H (J/g)
Procodile	14.67	−7.65	1.324	−11.06	21.21	2.436
ReziFlow	16.09	−13.9	2.279	−9.21	17.08	1.971
